# Comprehensive Characterization of Serum Lipids of Dairy Cows: Effects of Negative Energy Balance on Lipid Remodelling

**DOI:** 10.3390/metabo15040274

**Published:** 2025-04-15

**Authors:** Zhiqian Liu, Wenjiao Wang, Joanne E. Hemsworth, Coralie M. Reich, Carolyn R. Bath, Monique J. Berkhout, Muhammad S. Tahir, Vilnis Ezernieks, Leah C. Marett, Amanda J. Chamberlain, Mike E. Goddard, Simone J. Rochfort

**Affiliations:** 1 Agriculture Victoria Research, AgriBio, 5 Ring Road, Bundoora, VIC 3083, Australia; wwj224@126.com (W.W.); joanne.hemsworth@agriculture.vic.gov.au (J.E.H.); coralie.reich@agriculture.vic.gov.au (C.M.R.); carolyn.bath@agriculture.vic.gov.au (C.R.B.); sajid.tahir@agriculture.vic.gov.au (M.S.T.); vilnis.ezernieks@agriculture.vic.gov.au (V.E.); amanda.chamberlain@agriculture.vic.gov.au (A.J.C.); michael.goddard@agriculture.vic.gov.au (M.E.G.); simone.rochfort@agriculture.vic.gov.au (S.J.R.); 2 College of Horticulture, Shanxi Agricultural University, Jinzhong 030801, Shanxi, China; 3 Agriculture Victoria Research, Ellinbank Centre, Ellinbank, VIC 3821, Australia; monique.berkhout@agriculture.vic.gov.au (M.J.B.); leah.marett@agriculture.vic.gov.au (L.C.M.); 4 School of Agriculture and Food, Faculty of Veterinary and Agricultural Sciences, The University of Melbourne, Melbourne, VIC 3010, Australia; 5 School of Applied Systems Biology, La Trobe University, Bundoora, VIC 3083, Australia

**Keywords:** cows, serum, lipidomic analysis, transition period, negative energy balance

## Abstract

Background: The presence and concentration of lipids in serum of dairy cows have significant implications for both animal health and productivity and are potential biomarkers for several common diseases. However, information on serum lipid composition is rather fragmented, and lipid remodelling during the transition period is only partially understood. Methods: Using a combination of reversed-phase liquid chromatography-mass spectrometry (RP-LC-MS), hydrophilic interaction-mass spectrometry (HILIC-MS), and lipid annotation software, we performed a comprehensive identification and quantification of serum of dairy cows in pasture-based Holstein-Friesian cows. The lipid remodelling induced by negative energy balance was investigated by comparing the levels of all identified lipids between the fresh lactation (5–14 days in milk, DIM) and full lactation (65–80 DIM) stages. Results: We identified 535 lipid molecular species belonging to 19 classes. The most abundant lipid class was cholesteryl ester (CE), followed by phosphatidylcholine (PC), sphingomyelin (SM), and free fatty acid (FFA), whereas the least abundant lipids included phosphatidylserine (PS), phosphatidic acid (PA), phosphatidylglycerol (PG), acylcarnitine (AcylCar), ceramide (Cer), glucosylceramide (GluCer), and lactosylceramide (LacCer). Conclusions: A remarkable increase in most lipids and a dramatic decrease in FFAs, AcylCar, and DHA-containing species were observed at the full lactation compared to fresh lactation stage. Several serum lipid biomarkers for detecting negative energy balance in cows were also identified.

## 1. Introduction

Lipids play key roles in various biological functions, such as energy storage, cell membrane structure, and signalling. Lipid metabolism in dairy cows involves complex biochemical pathways and regulation mechanisms during the gestation–lactation cycle. The transition period (three weeks pre- to three weeks post-calving) is considered the most nutritionally and metabolically demanding phase during the lactation cycle. For cows reaching positive energy balance (around 45 days after calving), dietary fats are the principal source of essential lipids for various physiological functions and milk production; however, nearly all cows experience a negative energy balance (energy demands for milk production exceed dietary intake) during the fresh lactation period [1], and they also mobilize fat reserves from adipose tissue to compensate for reduced dry matter intake and to support milk production [2]. Lipids of different origins, either absorbed from the diet, of rumen bacterial origin (via fermentation and biohydrogenation), or through the mobilization of adipose tissue, are all transported via the bloodstream to different organs. Consequently, the balance and concentration of serum lipids can be indicative of the animal’s metabolic status, nutrient intake, and overall health [3].

Serum lipid profiles can be influenced by various factors such as diet, lactation stage, and health conditions [4,5]. Alterations in lipid concentration during the transition period from late gestation to fresh lactation have been the subject of numerous studies [6,7,8,9]. It is widely recognized that during the transition period, increased lipid mobilization from adipose tissue results in elevated plasma free fatty acid (FFA) and ketone body β-hydroxybutyric acid (BHBA) levels [10,11,12,13]. However, the response and remodelling of the entire lipidome of serum to negative energy balance in transition cows is unclear.

Despite the recognized importance of serum lipids for the health, milk production, and reproductive performance of dairy cows, information on detailed serum lipid composition of lactating cows has been limited. Early research established that bovine serum lipids primarily consist of triglycerides (TAGs), phospholipids, cholesteryl esters (CEs), and non-esterified fatty acids (NEFAs) or FFAs [14]. The advancement of analytical technologies especially liquid chromatography-mass spectrometry (LC-MS) in recent decades has enabled the identification of serum lipids at the species level. For example, Imhasly et al. [6] reported the changing pattern of five classes and 32 species of plasma lipid during the transition period, and Rico et al. [15] recently identified 301 species of plasma lipids belonging to three lipid categories (TAG, phospholipid, and CE). However, a comprehensive serum lipid inventory of dairy cows that covers all lipid classes is still lacking.

In this study, we first compared the FA profile of fresh and full lactation serum to unveil the overall lipid composition change during negative energy balance in a pasture-based system. We then conducted a comprehensive serum lipid identification and quantification at the two lactation stages with the aim to build a bovine serum lipid library and to dissect the detailed lipid remodelling pattern. Finally, we performed serum lipidomic profiling of individual cows from two large cohorts (143 cows in 2022 and 218 cows in 2023) to identify the most sensitive biomarkers that are associated with cows in negative energy balance.

## 2. Materials and Methods

### 2.1. Serum Sample Collection

This research was conducted at the Agriculture Victoria Research Ellinbank SmartFarm in Victoria, Australia (38°14′ S, 145°56′ E) with approval from the Department of Energy, Environment and Climate Action (DEECA) Agricultural Research and Extension Animal Ethics Committee (approval code: AEC 2022-04; approval date: 11 May 2022). All procedures were conducted in accordance with the Australian Code of Practice for the Care and Use of Animals for Scientific Purposes (NHMRC, 2013).

Cows were grazing pasture and supplemented twice daily with a grain mix determined by general farm practice throughout the lactation. Pasture on offer was predominantly perennial ryegrass and was allocated to cows at approximately 25 kg DM/cow per day. The grain mix was offered to individual cows at milking times in the dairy parlour and generally comprised wheat, barley, and canola meal and offered at amounts ranging from 6 to 8 kg/cow/day, depending on stage of lactation and pasture availability. Cows were milked twice daily at ~7:00 and 15:00.

Blood samples were collected immediately after morning milking from a cohort of 149 and 225 spring-calving (July to September) Holstein-Friesian cows in 2022 and 2023, respectively, at two lactation stages: fresh lactation (5–14 DIM) and full lactation (65–80 DIM). Each cohort comprised both primiparous heifers and multiparous cows (age: 2–11 years; parity: 1–9; average daily milk production: 33 L). Cows that were sampled in 2022 were not sampled again in 2023. The large sample size aimed to cover the inter-cow variation in response to negative energy balance. Blood sampling was conducted via coccygeal venipuncture into 10 mL vacutainers containing clotting activators for serum collection (BD Vacutainer System, Plymouth, UK). Samples were incubated at 25 °C for 30 min before being centrifuged at 1300× *g* at 25 °C for 10 min. Samples were then snap frozen in dry ice, transported to the laboratory and stored at −80 °C.

### 2.2. Chemicals

Mouse Splash^®^ Lipidomix standards (a mix of 14 deuterated standards), ceramide (Cer) Lipidomix standards, glucosylceramide (GluCer) and lactosylceramide (LacCer) standards were purchased from Avanti Lipids. The acylcarnitine (AcylCar), FFAs, and short-chain fatty acid (SCFA) and BHBA standards were from Sigma-Aldrich (St. Louis, MO, USA). Solvents used for serum lipid extraction and mobile phase preparation were of chromatographic or LC-MS grade. Methanol and isopropanol were from Fisher Scientific, chloroform from Sigma-Aldrich, acetonitrile and butanol from Merck, and acetonitrile containing 0.1% formic acid from Fisher Chemical. Ammonium formate (a mobile phase additive) was of analytical grade (Sigma-Aldrich).

### 2.3. Sample Preparation for LC-MS and GC Analysis

For the comparison of the FA profile (between the two lactation stages) and the construction of bovine serum lipid library, pooled serum samples (15 cows randomly selected from the 2022 cohort, i.e., around 10% of the population) were analysed. Serum lipids were extracted either by the Folch method [16] using chloroform/methanol (2:1, *v*/*v*) (for FA analysis by GC), or by the one-phase method using a solvent mix composed of butanol, methanol, and chloroform (at a 3:5:4 ratio) [17], after spiking the internal standards (IS), i.e., deuterated Mouse Splash Lipidomix standard mix (for lipid identification and quantification by LC-MS).

For the search of lipid biomarkers that differentiate fresh and full lactation stages, all individual serum samples (143 for the 2022 cohort and 218 for the 2023 cohort) were extracted by the one-phase method prior to LC-MS analysis.

### 2.4. Identification of Serum Lipids by RP-LC-MS/MS

The total lipids extracted from the pooled serum were separated by a Kinetex C18 column (100 × 2.1 mm, 1.7 µm, Phenomenex) on a Vanquish UHPLC system (Thermo Fisher Scientific, Waltham, MA, USA). The column compartment was maintained at 50 °C and the sample tray at 12 °C. The mobile phase is composed of water/acetonitrile (40:60, *v*/*v*) containing 10 mM ammonium formate (A) and acetonitrile/isopropanol (10:90, *v*/*v*) containing 10 mM ammonium formate (B). The gradient elution was performed by a linear increase in mobile phase B from 32 to 97% over 21 min with a flowrate of 0.25 mL/min.

A Q Exactive Plus mass spectrometer (Thermo Fisher Scientific) equipped with a heated electrospray ionization (HESI) source was used for the detection of all lipid classes. The heated capillary was maintained at 300 °C with a source heater temperature of 300 °C, and the sheath, auxiliary and sweep gases were at 30, 10 and 0 units, respectively. The instrument was operated in either positive (4.2 kV) or negative (3.6 kV) ion mode with a full scan (120–1600 *m*/*z*) at a resolution of 70,000 followed by 15 data-dependent MS2 scans at a resolution of 17,500 and collision energy of 25 eV. The precursor isolation width was set to 1.5 Da and a dynamic exclusion of 7 s was enabled. Lipid identification at the species level was achieved using the LipidSearch 4.1 software package (Thermo Fisher Scientific), based on the accurate-mass information of parent ions as well as MS2 spectra. The detailed parameters selected were reported previously [18].

### 2.5. Quantification of Serum Lipids

For the construction of serum lipid library, TAGs, CEs, and FFAs were quantified by RP-LC-MS using the same LC settings as described in the previous section. In the case of SCFA, a derivatization step was performed prior to the RP-LC-MS analysis [19]. For the quantification of all other lipid classes, HILIC-MS was employed. The LC separation was achieved on the same Vanquish UHPLC system with a HILIC column (150 × 4.6 mm, 2.6 µm, Phenomenex) maintained at 30 °C. The mobile phase comprised 10 mM ammonium formate (A) and acetonitrile containing 0.1% formic acid (B). The gradient elution was performed by a linear increase in mobile phase A from 2 to 40% over 16 min with a flowrate of 0.5 mL/min.

The same Q Exactive MS detector was employed for lipid quantification based on parent ion scan acquired simultaneously in positive and negative mode. The concentration of PC, PE, PI, SM, LPC, LPE, PCP, PEP, TAG, and CE species was calculated using the one-point calibration method—i.e., concentration of a lipid species = (peak area of the lipid species/peak area of the IS) × concentration of the IS—whereas that of all other lipids (GluCer, LacCer, Cer, AcylCar, FFAs, and SCFAs), was determined by the external calibration method. The content of each lipid class is estimated by the sum of all species within the same class.

For lipidomic biomarker identification for cows in negative or positive energy balance, 143 (from 2022 cohort) and 218 cows (from 2023 cohort) with 2 time point serum samples were included, whereas 6 (from 2022 cohort) and 7 cows (from 2023 cohort) with only one time point serum samples were excluded. The serum samples were analysed in randomized order using a higher-throughput HILIC-MS method with a total runtime of 16 min. The relative abundance (peak area) of all lipid species was used for a chemometrics analysis. The MS response fluctuation was monitored by injecting the Mouse Splash Lipidomix standard mix after every 20 samples.

### 2.6. Data Analysis

Statistical comparison between pooled samples of the fresh and full lactation stages was conducted by Student’s *t*-test. A chemometrics analysis of lipid data was carried out with MetaboAnalyst 6.0 (http://www.metaboanalyst.ca, accessed on 18 August 2024) [20], after pre-processing the raw data by autoscaling.

## 3. Results

### 3.1. FA Composition of Bovine Serum

**Figure 1:**
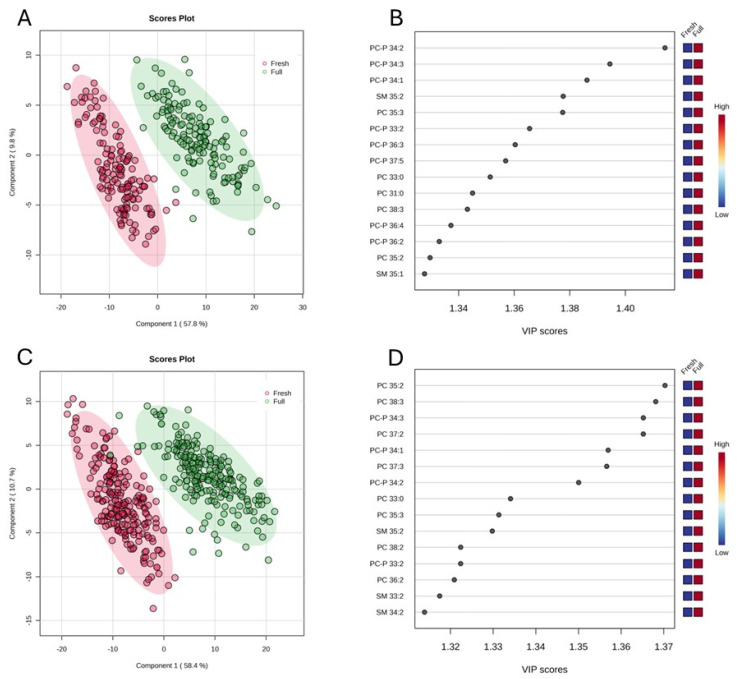
PLS-DA plot of serum samples collected at fresh lactation (Fresh) and full lactation (Full) stages in 2022 (*n* = 143) (**A**) and 2023 (*n* = 218) (**C**), and the VIP scores of the top 15 features differentiating the two lactation stages for 2022 (**B**) and 2023 (**D**) cohorts.

Bovine serum lipids are featured by the presence of five major FAs (C16:0, C18:0, C18:1c9, C18:2c9,12, and C18:3n3), four intermediate FAs (C16:1, C20:3, C20:4, and C20:5), and a number of minor yet quantifiable FAs (C18:1t11, C18:2c9t11, C15:0, C15:0-anteiso, C17:0, and so on) (Figure 1). In addition, a quasi-absence of FAs shorter than C14 was observed in bovine serum.

A significant increase in the concentration of most FAs was observed from fresh lactation to full lactation. Conversely, the concentration of C18:1t11, C18:2c9t11 (a conjugated linoleic acid or CLA) and C22:6n3 (DHA) decreased from fresh lactation to full lactation (Table 1). The total serum lipid content, measured as the sum of all FAs, nearly doubled from fresh lactation to full lactation (Table 1).

The percentage of individual FAs also fluctuated between the two stages of lactation and the direction of change varied with FA species. For example, among the major FAs, a substantial reduction in the proportion of C16:0 and C18:1c9 and a concomitant increase in the proportion of C18:2c9,12 and C18:3n3 was observed at full lactation as compared to fresh lactation (Table 1). A remarkable increase in the proportion of FAs was also observed with lower abundance species such as C15:0, C15:0-iso, C15:0-anteiso, and C17:0 (Table 1).

### 3.2. Lipidomic Composition of Bovine Serum

Using tandem RP-LC-MS and LipidSearch software, we identified a total 350 lipid groups (a lipid group is defined as a series of lipid species or isomers sharing the same chemical formula and accurate mass but differing in FA composition) and 535 lipid molecular species in the serum of dairy cows. These lipids belong to 19 classes, but around half of the species were found in four classes PC, TAG, SM, and PE (Table 2), whereas only a small number (<10) of species were detected within PS, PA, PG, and FFA classes. As expected, TAGs contained more FA compositional isomers, but 2–3 isomers were also detected with many PC and SM groups (Table 2).

The quantification of the lipids was performed by a combination of RP-LC-MS, off-line derivatization followed by RP-LC-MS and HILIC-MS. The isomer species cannot be separated by RP-LC or HILIC, so they were quantified as a group. The most abundant lipid classes of serum were CE, PC, SM, PCP, and LPC, with concentrations reaching 5672, 1272, 261, 237, and 114 µg/mL, respectively, at the full lactation stage; the most abundant species were CE 18:2, CE 18:3, PC 36:2, and PC 34:2. The least abundant lipid classes at the same lactation stage were GluCer, LacCer, Cer, PS, and AcylCar, the concentrations of which were only 0.39, 1.23, 0.79, 0.39, and 0.24 µg/mL, respectively. With a total concentration of 70.2 and 51.7 µg/mL, respectively, TAGs and FFAs were present at an intermediate level in serum. The full list of molecular species identified in each class and their concentration at the two lactation stages are summarized in Table 2.

The results in Table 2 indicate that the lipid content of serum varied substantially with lactation stage. At the class level, a higher concentration (>20%) was observed at the full lactation stage for 16 out of the 19 classes. Only two classes, AcylCar and FFAs, displayed an opposing trend (higher concentration at the post-calving stage), whereas little difference was observed for PS. The greatest fold change was observed for PCP and FFAs, followed by PC, AcylCar, SM, and PEP (Table 2).

At the species (group) level, most lipids within the 16 classes exhibited a substantial rise in concentration from fresh lactation to full lactation, with the only exceptions being PI 40:6, PEP 40:6, PE 40:6, PC 40:7, PC 40:6, and LPC 22:6, which underwent a significant drop with the progression of lactation. By contrast, all species of FFAs and most AcylCar species (except Car 3:0/Car 5:0) showed a drastic decrease from fresh lactation to full lactation (Table 2).

### 3.3. Serum Lipid Biomarkers Differentiating the Two Lactation Stages

The results obtained with pooled samples from the 2022 cohort (shown in Table 2) provide a gross picture of the lipidomic change in relation to lactation stage. The pattern revealed was further verified by an analysis of all individuals from the 2022 and 2023 cohorts. A total of 198 polar lipid and FFA features captured by the higher-throughput HILIC-MS workflow were subjected to chemometric analysis. Figure 1A,C show the clear separation by PLS-DA of samples by lactation stages for both cohorts. The top 15 most differential lipid species (ranked by the importance of variables in the model) for each cohort are shown in Figure 1B,D; 9 out of the 15 lipids (PCP 34:1, PC-P 34:2, PCP 34:3, PCP 33:2, PC 33:0, PC 35:2, PC 35:3, PC 38:3, and SM 35:2) were common across the two cohorts. The contrasting differences in the top 5 lipids between the two lactation stages are illustrated in Figure S1 (Supplementary Materials).

**Figure 2:**
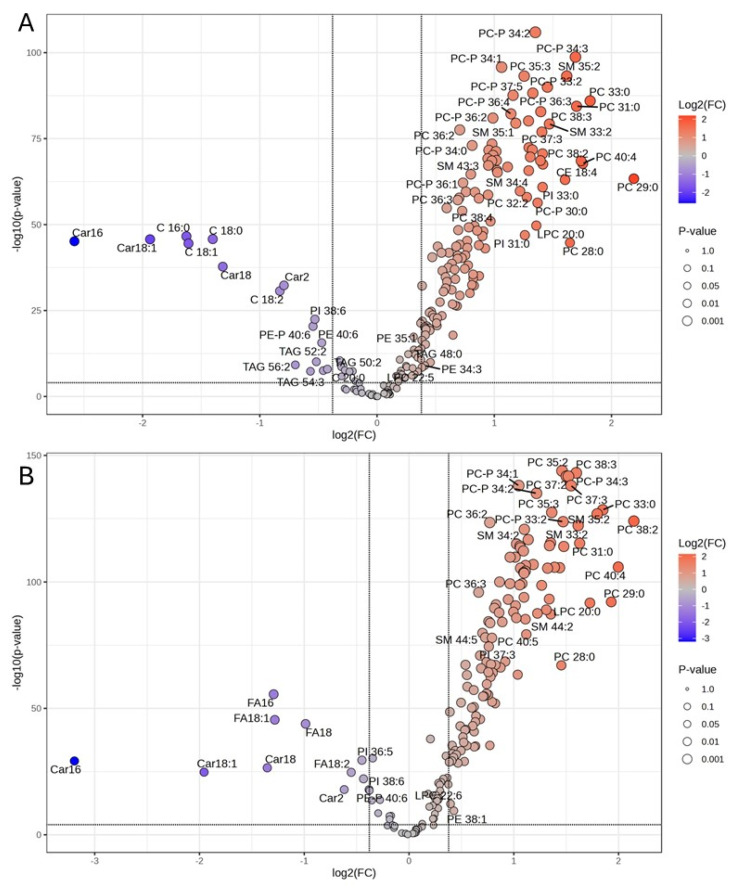
Volcano plot of upregulated and downregulated (full lactation vs. fresh lactation) lipid species in serum of 2022 cohort (**A**) and 2023 cohort (**B**) (*p* < 0.0001 and fold change > 1.3).

This large cohort validation analysis also confirmed the widespread shift in serum lipid level during the transition period. For both 2022 and 2023 cohorts, over 92% of the lipid features detected showed a significant change between the two lactation stages (*p* < 0.05). Although the top 15 most differential lipids were all upregulated at the full lactation stage (or in positive energy balance), volcano plots that consider both *p* value (<0.0001) and fold change (>1.3) also allowed the identification of 16 and 11 upregulated lipids at the fresh lactation period (or in negative energy balance) for 2022 and 2023 cohorts, respectively (Figure 2A,B). While upregulated species at the full lactation are in large numbers and mostly from PCP and PC classes, upregulated lipids at the fresh lactation were mainly FFAs (FA 16:0, FA 18:0, FA 18:1, and FA 18:2) and Acylcar (Car 2:0, Car 16:0, Car 18:0, and Car 18:1) (Figure 2A,B).

## 4. Discussion

Compared to the abundant data on milk lipids, much less is known about the serum lipids of dairy cows and the remodelling of all lipid categories during negative energy balance. Our study aimed to fill this gap. The total FA content in serum was found to be about 10-fold lower than typically present in milk, and only five major FAs were detected in serum compared to a dozen major FAs in milk [21]. Another interesting feature of the serum FA profile is the much higher level (>60%) of unsaturated FAs compared to milk. This may be because serum lipids are mostly derived from plant-based feeds, which contain a high proportion of unsaturated FAs. On the other hand, judging by the relatively low levels of the intermediates of biohydrogenation C18:1t11 and C18:2c9t11, the process of bacterial biohydrogenation does not appear to drastically change the FA makeup in the rumen.

Most FAs showed a dramatic increase (up to 3-fold) from fresh lactation to full lactation. This likely results from increased dry matter intake and enhanced microbial activities in the rumen as dairy cows transition from negative to positive energy balance, because both plant-derived unsaturated FAs (such as C18:1, C18:2, and C18:3) and rumen bacteria-generated branched/odd-chain FAs (C15:0, C15:0-iso and C17:0) were augmented [22]. By contrast, C22:6n3 (DHA), C18:1t11, and CLAc9t11 declined from fresh lactation to full lactation. DHA can be obtained from the feed or synthesized from alpha-linolenic acid (ALA; 18:3n-3) in the liver [23]. Thus, a higher level of DHA in the serum at fresh lactation suggests that its biosynthesis may be upregulated during the transition period, given that a non-DHA-rich diet supplement was provided to cows after calving. As for C18:1t11 and CLAc9t11, both are intermediates of biohydrogenation in the rumen. The reason for the reduced biohydrogenation activity at full lactation remains to be determined.

The relative proportion (or %) of the FA also varied with lactation stage. The most striking observation is the significant decline of the proportion of C16:0 and C18:1 and the concomitant increase in the proportion of C18:2 and C18:3 at the full lactation stage compared to fresh lactation stage. This shift in FA proportion can be attributed to the reduced mobilization of adipose tissue [24], and increased intake of plant-based lipids as dairy cows recover from negative energy balance.

The number of TAG species detected in bovine serum is far smaller than previously reported for bovine milk [25]. This is because the number of TAG species is solely determined by the FA diversity in a biological system. While both preformed FA (odd-chain and long-chain FA derived from the blood) and de novo synthesized FA (C4:0 to C16:0) are used for lipid synthesis in the mammary gland [26], only plant-based FA and rumen bacteria-generated FA are available for serum lipid synthesis. It is worth mentioning that the number of lipids identified is not exhaustive, and more low-abundance species will be detected by sample enrichment or employing more sensitive LC-MS settings (e.g., nano-LC-MS).

A close examination of the lipid structure vs. concentration revealed that regardless of lipid class, the most abundant species are those containing one or more of the five major FAs (C18:2, C18:1, C18:3, C16:0, and C18:0). We should point out that the number of FAs found in the lipid structures (Table 2) is far greater than that revealed by GC in FA profiling (Table 1). This is firstly because LC-MS is more sensitive than GC-FID, and secondly, the presence of a particular FA can be deduced from tandem LC-MS spectrum, whereas the identity of FAs cannot be confirmed without standards in GC-FID analysis.

In this study, the results obtained with pooled serum samples provide a detailed account of lipid remodelling under negative energy balance. A widespread and dramatic suppression of nearly all lipid classes in the serum of post-calving cows is an important finding. The lower level of most lipid classes at the fresh lactation stage is consistent with the global FA profiling data (Table 1), implying that a shortage in FA supply may be responsible for the decline in circulating lipids, synthesized in the liver and/or in the intestine enterocytes. The higher level of FFAs in the serum during negative energy balance observed in this study is consistent with the literature. This is believed to be caused by adipose tissue mobilization, and some FFA species have been found to have high predictive power for the diagnosis of ketosis in early lactating cows [27,28]. However, in this study, a higher level of FFAs was accompanied by only a slightly greater concentration of BHBA in the serum of cows at the early lactating stage. Thus, the quantitative relationship between FFAs and BHBA remains unclear.

During negative energy balance we observed also an increase in AcylCar and DHA-containing species. Unlike FFAs, the exact mode of function of AcylCar in lipid metabolism during negative energy balance is not well understood. The main function of AcylCar is the transport of FAs to mitochondria for oxidation [29]. Therefore, the increase in AcylCar in the serum of cows in the transition period may result from reduced lipid oxidation activity in the mitochondria in the liver cells [30]. Our findings are in accordance with observations of Ghaffari et al. [31], who found a greater concentration of circulating acetylcarnitine in the serum of cows during fresh lactation. Moreover, increased plasma concentrations of AcylCar have been suggested as a marker of metabolism disorders [32]. Although the role of DHA in transition cow’s health remains unclear. It is plausible that the enhanced production of DHA has a positive effect on the immune function and health status of dairy cows during this critical period [33]. It is also possible that more DHAs are synthesized in the liver and circulated in the blood in the fresh lactation stage to promote brain development of neonates [34].

The results obtained with pooled serum samples (Table 1 and Table 2) cannot unveil the variation across animals nor allow the discovery of robust lipid biomarkers between the two lactation stages. Lipidomic profiling was thus performed on large cohorts of 2022 and 2023, and a multivariate classification analysis (PLS-DA) of the large dataset revealed the contrasting lipid makeup between the two lactation stages. Overall, the lipidomic response to negative energy balance was similar between the 2022 and the 2023 cohorts. The slight year-to-year discrepancy in the top 15 lipids differentiating the two lactation stages may result from environmentaland dietary variation across the two years. Taken together, a number of PCP, PC, and SM species were the top downregulated markers, whereas several FFAs, Acylcar, and DHA-containing phospholipid species the best upregulated markers of negative energy balance in dairy cows. It is worth noting that several PCP species were among the top and reproducible markers. Plasmalogens are thought to be involved in the membrane bilayer formation and possess an antioxidant function [35]. The rise in PCP levels at the full lactation stage may be an indicator of recovery from a negative energy balance.

## 5. Conclusions

We have revealed the overwhelming change in both polar and nonpolar lipids of serum during transition period and identified several upregulated and downregulated biomarkers that distinguish the fresh lactating and full lactating cows. To our knowledge, this is the most comprehensive survey on serum lipids and lipidomic remodelling of dairy cows suffering from negative energy balance. Our findings contribute to a better understanding of the lipid metabolism in dairy cows during the transition period. In addition, both the lipid structure and concentration presented in this paper may serve as a useful reference for researchers of dairy and veterinary science.

## Figures and Tables

**Table 1:** Fatty acid composition of bovine serum at fresh and full lactation stages.

FA	Concentration (µg/mL)		%	
	Fresh Lactation	Full Lactation	Fresh Lactation	Full Lactation
C14:0	9.06 ^b^	16.68 ^a^	0.49	0.49
C15:0-iso	5.26 ^b^	16.12 ^a^	0.29	0.48
C15:0-anteiso	8.46 ^b^	28.12 ^a^	0.46	0.83
C15:0	10.74 ^b^	25.62 ^a^	0.59	0.76
C16:0	236.32 ^b^	336.82 ^a^	12.90	9.98
C16:1	91.50 ^b^	171.38 ^a^	5.00	5.08
C17:0-iso	8.84 ^b^	15.02 ^a^	0.48	0.44
C17:0	13.62 ^b^	31.3 ^a^	0.74	0.93
C18:0	297.84 ^b^	494.06 ^a^	16.26	14.64
C18:1t11	38.80 ^a^	26.94 ^b^	2.12	0.80
C18:1c9	271.74 ^b^	336.8 ^a^	14.83	9.98
C18:1c11	17.66 ^b^	21.62 ^a^	0.96	0.64
C18:2c9,12	540.20 ^b^	1265.12 ^a^	29.49	37.48
C18:2c9t11	9.26 ^a^	5.48 ^b^	0.51	0.16
C18:3n6	6.28 ^b^	29.02 ^a^	0.34	0.86
C18:3n3	172.52 ^b^	378.2 ^a^	9.42	11.20
C20:3n6	19.54 ^b^	65.52 ^a^	1.07	1.94
C20:4n6	38.52 ^b^	60.84 ^a^	2.10	1.80
C20:5n3	27.82 ^b^	47.48 ^a^	1.52	1.41
C22:6n3	7.80 ^a^	3.18 ^b^	0.43	0.09
Sum	1831.78 ^b^	3375.32 ^a^	100.00	100.00

Note: Values are means of 3 measurements of pooled samples from 15 cows. Within each row, fatty acid concentrations followed by different letters are significantly different between the two lactation stages (*p* < 0.05).

**Table 2:** List of lipid species identified in the serum of dairy cows and their concentrations (µg/mL) at two lactation stages.

Lipid Species	Formula	*m*/*z*	Isomer 1	Isomer 2	Isomer 3	Isomer 4	Fresh Lactation	Full Lactation
TAG		[M+NH_4_]^+^						
TAG26:0	C_29_H_54_O_6_	516.4264	(8:0_8:0_10:0)				0.2064 ± 0.0315	0.2965 ± 0.0160
TAG34:0	C_37_H_70_O_6_	628.5516	(16:0_8:0_10:0)	(4:0_14:0_16:0)			0.0431 ± 0.0013	0.0695 ± 0.0012
TAG38:0	C_41_H_78_O_6_	684.6142	(4:0_16:0_18:0)	(6:0_14:0_18:0)	(12:0_12:0_14:0)	(6:0_16:0_16:0)	0.0305 ± 0.0009	0.0355 ± 0.0044
TAG42:1	C_45_H_84_O_6_	738.6612	(16:0_8:0_18:1)	(16:1_12:0_14:0)	(18:1_12:0_12:0)		0.0213 ± 0.0007	0.0321 ± 0.0050
TAG43:1	C_46_H_86_O_6_	752.6768	(15:0_14:0_14:1)	(16:1_13:0_14:0)	(15:0_12:0_16:1)		0.0337 ± 0.0032	0.0518 ± 0.0048
TAG44:1	C_47_H_88_O_6_	766.6925	(16:0_12:0_16:1)	(16:0_14:0_14:1)	(16:1_14:0_14:0)		0.1118 ± 0.0051	0.1915 ± 0.0329
TAG45:2	C_48_H_88_O_6_	778.6925	(15:1_14:0_16:1)	(15:0_14:1_16:1)			0.0509 ± 0.0016	0.0804 ± 0.0168
TAG45:1	C_48_H_90_O_6_	780.7081	(15:0_14:0_16:1)				0.1758 ± 0.0054	0.3040 ± 0.0481
TAG45:0	C_48_H_92_O_6_	782.7238	(15:0_14:0_16:0)				0.2575 ± 0.0123	0.4781 ± 0.0515
TAG46:2	C_49_H_90_O_6_	792.7081	(16:0_14:1_16:1)	(16:1_14:0_16:1)			0.1645 ± 0.0018	0.2671 ± 0.0463
TAG46:1	C_49_H_92_O_6_	794.7238	(16:0_14:0_16:1)				0.4374 ± 0.0276	0.6411 ± 0.0712
TAG46:0	C_49_H_94_O_6_	796.7394	(16:0_14:0_16:0)	(15:0_15:0_16:0)			0.4742 ± 0.0071	0.7479 ± 0.0700
TAG47:2	C_50_H_92_O_6_	806.7238	(15:1_16:0_16:1)				0.1390 ± 0.0094	0.2965 ± 0.0561
TAG47:1	C_50_H_94_O_6_	808.7394	(15:0_16:0_16:1)	(15:1_16:0_16:0)			0.4598 ± 0.0281	0.8254 ± 0.1136
TAG47:0	C_50_H_96_O_6_	810.7551	(15:0_16:0_16:0)				0.5620 ± 0.0187	1.1085 ± 0.0521
TAG48:2	C_51_H_94_O_6_	820.7394	(16:1_14:0_18:1)	(16:0_16:1_16:1)	(17:0_16:1_15:1)		0.5784 ± 0.0187	0.7987 ± 0.0791
TAG48:1	C_51_H_96_O_6_	822.7551	(16:0_14:0_18:1)	(16:0_16:0_16:1)	(15:0_15:0_18:1)		0.9912 ± 0.0349	1.3890 ± 0.0894
TAG48:0	C_51_H_98_O_6_	824.7707	(18:0_16:0_14:0)	(16:0_16:0_16:0)	(15:0_16:0_17:0)		1.0444 ± 0.0338	1.8805 ± 0.0534
TAG49:2	C_52_H_96_O_6_	834.7551	(16:0_16:1_17:1)	(15:0_16:1_18:1)			0.5600 ± 0.0128	0.8307 ± 0.0580
TAG49:1	C_52_H_98_O_6_	836.7707	(15:0_16:0_18:1)	(15:1_16:0_18:0)	(16:0_16:0_17:1)		0.9626 ± 0.0106	1.7950 ± 0.0561
TAG49:0	C_52_H_100_O_6_	838.7864	(18:0_15:0_16:0)				1.0198 ± 0.0232	2.2705 ± 0.0046
TAG50:3	C_53_H_96_O_6_	846.7551	(16:1_16:1_18:1)	(16:0_16:1_18:2)			0.6908 ± 0.0128	0.7933 ± 0.0240
TAG50:2	C_53_H_98_O_6_	848.7707	(16:0_16:1_18:1)				1.6146 ± 0.0359	1.6401 ± 0.0534
TAG50:1	C_53_H_100_O_6_	850.7864	(16:0_16:0_18:1)	(15:0_17:0_18:1)	(18:0_16:0_16:1)		2.7591 ± 0.0545	3.3550 ± 0.0740
TAG51:3	C_54_H_98_O_6_	860.7707	(15:0_18:1_18:2)	(16:0_17:1_18:2)			0.3803 ± 0.0108	0.5823 ± 0.0258
TAG51:2	C_54_H_100_O_6_	862.7864	(15:0_18:1_18:1)	(15:1_18:0_18:1)	(16:0_17:1_18:1)		1.0382 ± 0.0094	1.5439 ± 0.0167
TAG51:1	C_54_H_102_O_6_	864.8020	(16:0_16:1_19:0)	(15:1_16:0_20:0)	(18:0_15:0_18:1)	(18:0_16:0_17:1)	1.8230 ± 0.0375	3.2108 ± 0.0534
TAG52:4	C_55_H_98_O_6_	872.7707	(16:1_18:1_18:2)	(16:0_18:1_18:3)			0.8114 ± 0.0071	0.8361 ± 0.0245
TAG52:3	C_55_H_100_O_6_	874.7864	(16:1_18:1_18:1)	(18:0_16:0_18:3)	(16:0_18:1_18:2)		2.1275 ± 0.0106	2.1129 ± 0.0245
TAG52:2	C_55_H_102_O_6_	876.8020	(16:0_18:1_18:1)				4.6802 ± 0.0716	4.5410 ± 0.0682
TAG52:1	C_55_H_104_O_6_	878.8177	(18:0_16:0_18:1)	(18:0_16:1_18:0)	(20:0_16:0_16:1)		5.9208 ± 0.0962	7.9334 ± 0.1244
TAG52:0	C_55_H_106_O_6_	880.8333	(20:0_16:0_16:0)	(18:0_16:0_18:0)			5.0501 ± 0.3361	7.4179 ± 0.0303
TAG53:2	C_56_H_104_O_6_	890.8177	(18:0_17:1_18:1)	(19:1_16:1_18:0)	(17:0_18:1_18:1)		0.9258 ± 0.0061	1.1486 ± 0.0167
TAG53:1	C_56_H_106_O_6_	892.8333	(18:0_15:0_20:1)	(18:0_17:0_18:1)	(20:0_15:0_18:1)	(18:0_17:1_18:0)	1.5635 ± 0.0591	1.9339 ± 0.0303
TAG54:4	C_57_H_102_O_6_	900.8020	(18:1_18:1_18:2)	(18:0_18:1_18:3)			1.5737 ± 0.0128	1.6214 ± 0.0122
TAG54:3	C_57_H_104_O_6_	902.8177	(18:0_18:1_18:2)	(18:1_18:1_18:1)			3.3415 ± 0.0479	3.1760 ± 0.0303
TAG54:2	C_57_H_106_O_6_	904.8333	(18:0_16:1_20:1)	(18:0_18:0_18:2)	(18:0_18:1_18:1)		4.6638 ± 0.1382	4.8963 ± 0.1319
TAG54:1	C_57_H_108_O_6_	906.8490	(17:0_18:1_19:0)	(18:0_18:0_18:1)	(20:0_16:0_18:1)		6.2764 ± 0.1178	6.5658 ± 0.1091
TAG55:2	C_58_H_108_O_6_	918.8490	(17:0_18:1_20:1)	(19:1_18:0_18:1)	(20:0_17:1_18:1)		0.3705 ± 0.0074	0.3953 ± 0.0046
TAG55:1	C_58_H_110_O_6_	920.8646	(19:1_18:0_18:0)	(18:0_17:1_20:0)	(18:0_16:0_21:1)		0.5794 ± 0.0195	0.8655 ± 0.0910
TAG56:3	C_59_H_108_O_6_	930.849	(18:0_18:2_20:1)	(18:0_18:0_20:3)	(20:0_18:1_18:2)		0.6088 ± 0.0069	0.4888 ± 0.0000
TAG56:2	C_59_H_110_O_6_	932.8646	(18:0_18:1_20:1)	(18:0_18:2_20:0)	(20:0_18:1_18:1)		1.1445 ± 0.0154	0.7666 ± 0.0122
Sum TAG							56.2682 ± 0.1737 ^b^	70.2155 ± 0.9153 ^a^
PI		[M-H]^−^						
PI31:0	C_40_H_77_O_13_P	795.5024	(16:0_15:0)				0.0071 ± 0.0004	0.0238 ± 0.0016
PI32:1	C_41_H_77_O_13_P	807.5024	(18:1_14:0)	(16:0_16:1)			0.0386 ± 0.0006	0.0464 ± 0.0011
PI32:0	C_41_H_79_O_13_P	809.5180	(16:0_16:0)				0.0402 ± 0.0013	0.1105 ± 0.0021
PI33:1	C_42_H_79_O_13_P	821.5180	(15:0_18:1)	(17:1_16:0)			0.0542 ± 0.0008	0.1198 ± 0.0029
PI33:0	C_42_H_81_O_13_P	823.5337	(18:0_15:0)	(16:0_17:0)			0.0533 ± 0.0015	0.2338 ± 0.0097
PI34:3	C_43_H_77_O_13_P	831.5024	(16:0_18:3)				0.0555 ± 0.0012	0.0647 ± 0.0036
PI34:2	C_43_H_79_O_13_P	833.5180	(16:0_18:2)				0.3456 ± 0.0048	0.4018 ± 0.0131
PI34:1	C_43_H_81_O_13_P	835.5337	(16:0_18:1)				0.8015 ± 0.0146	1.0978 ± 0.0188
PI34:0	C_43_H_83_O_13_P	837.5493	(18:0_16:0)				0.0428 ± 0.0080	0.2710 ± 0.0118
PI35:3	C_44_H_79_O_13_P	845.5180	(17:0_18:2)				0.0277 ± 0.0006	0.0534 ± 0.0009
PI35:2	C_44_H_81_O_13_P	847.5337	(17:1_18:1)	(17:0_18:2)			0.1559 ± 0.0021	0.2925 ± 0.0088
PI35:1	C_44_H_83_O_13_P	849.5493	(17:0_18:1)	(17:1_18:0)	(19:1_16:0)		0.3010 ± 0.0027	0.6111 ± 0.0175
PI36:5	C_45_H_77_O_13_P	855.5024	(16:0_20:5)	(18:3_18:2)			0.0369 ± 0.0026	0.0356 ± 0.0003
PI36:4	C_45_H_79_O_13_P	857.5180	(16:0_20:4)	(18:3_18:1)			0.2985 ± 0.0043	0.3093 ± 0.0016
PI36:3	C_45_H_81_O_13_P	859.5337	(18:0_18:3)				1.2988 ± 0.0141	1.6947 ± 0.0436
PI36:2	C_45_H_83_O_13_P	861.5493	(18:1_18:1)	(18:0_18:2)			3.9503 ± 0.0373	5.3445 ± 0.1207
PI36:1	C_45_H_85_O_13_P	863.5650	(18:0_18:1)				3.9427 ± 0.0164	6.1799 ± 0.1146
PI37:4	C_46_H_81_O_13_P	871.5337	(17:0_20:4)	(18:4_19:0)	(18:0_19:4)		0.1533 ± 0.0059	0.2461 ± 0.0074
PI37:3	C_46_H_83_O_13_P	873.5493	(19:1_18:2)	(17:0_20:3)			0.1295 ± 0.0051	0.3232 ± 0.0072
PI37:2	C_46_H_85_O_13_P	875.5650	(18:0_19:2)				0.1367 ± 0.0047	0.2113 ± 0.0082
PI38:6	C_47_H_79_O_13_P	881.5180	(18:2_20:4)	(18:1_20:5)			0.1316 ± 0.0035	0.0825 ± 0.0018
PI38:5	C_47_H_81_O_13_P	883.5337	(18:0_20:5)	(18:1_20:4)			2.0842 ± 0.0543	2.0779 ± 0.0255
PI38:4	C_47_H_83_O_13_P	885.5493	(18:0_20:4)	(18:1_20:3)			6.1972 ± 0.1100	7.9707 ± 0.1304
PI38:3	C_47_H_85_O_13_P	887.5650	(18:0_20:3)	(18:1_20:2)			2.2851 ± 0.0220	6.7939 ± 0.1350
PI38:2	C_47_H_87_O_13_P	889.5806	(20:1_18:1)	(18:0_20:2)			0.1915 ± 0.0300	0.1895 ± 0.0341
PI38:1	C_47_H_89_O_13_P	891.5963	(18:0_20:1)				0.0477 ± 0.0028	0.0967 ± 0.0122
PI40:6	C_49_H_83_O_13_P	909.5493	(18:1_22:5)	(18:0_22:6)			0.2969 ± 0.0080	0.1908 ± 0.0043
PI40:5	C_49_H_85_O_13_P	911.5650	(18:0_22:5)				1.2850 ± 0.0145	1.8165 ± 0.0320
Sum PI							24.3982 ± 0.2140 ^b^	36.9094 ± 0.6746 ^a^
PEP		[M+H]^+^						
PEP32:2	C_37_H_70_O_7_NP	672.4968	(14:0p_18:2)				0.3346 ± 0.0139	0.8911 ± 0.0015
PEP32:1	C_37_H_72_O_7_NP	674.5124	(14:0p_18:1)	(16:0p_16:1)			0.1649 ± 0.0081	0.2143 ± 0.0001
PEP33:2	C_38_H_72_O_7_NP	686.5124	(16:1p_17:1)				0.8875 ± 0.0331	2.8026 ± 0.0175
PEP33:1	C_38_H_74_O_7_NP	688.5281	(14:0p_19:1)				0.3250 ± 0.0119	0.5116 ± 0.0077
PEP34:3	C_39_H_72_O_7_NP	698.5124	(16:0p_18:3)	(16:1p_18:2)	(14:0p_20:3)		0.7283 ± 0.0233	1.7172 ± 0.0176
PEP34:2	C_39_H_74_O_7_NP	700.5281	(16:0p_18:2)				1.1702 ± 0.0350	2.6059 ± 0.0337
PEP34:1	C_39_H_76_O_7_NP	702.5437	(20:0e_14:1)	(16:0p_18:1)			0.2892 ± 0.0095	0.3929 ± 0.0068
PEP35:2	C_40_H_76_O_7_NP	714.5437	(20:0p_15:2)				0.2728 ± 0.0048	0.6118 ± 0.0077
PEP36:5	C_41_H_72_O_7_NP	722.5124	(16:1p_20:4)	(16:0p_20:5)			0.2612 ± 0.0105	0.4530 ± 0.0093
PEP36:4	C_41_H_74_O_7_NP	724.5281	(16:0p_20:4)				0.3795 ± 0.0142	0.7764 ± 0.0036
PEP36:3	C_41_H_76_O_7_NP	726.5437	(18:0p_18:3)	(18:1p_18:2)	(16:0p_20:3)		0.4065 ± 0.0102	0.9758 ± 0.0144
PEP36:2	C_41_H_78_O_7_NP	728.5594	(18:0p_18:2)	(18:1p_18:1)			0.3095 ± 0.0088	0.4852 ± 0.0112
PEP36:1	C_41_H_80_O_7_NP	730.5750	(18:0p_18:1)				0.1386 ± 0.0034	0.1809 ± 0.0048
PEP38:5	C_43_H_76_O_7_NP	750.5437	(18:0p_20:5)				0.4181 ± 0.0134	0.4003 ± 0.0049
PEP38:4	C_43_H_78_O_7_NP	752.5594	(18:0p_20:4)				0.1720 ± 0.0052	0.2688 ± 0.0030
PEP38:1	C_43_H_84_O_7_NP	758.6063	(20:0p_18:1)				0.0014 ± 0.0011	0.0013 ± 0.0007
PEP40:6	C_45_H_78_O_7_NP	776.5594	(18:0p_22:6)				0.0777 ± 0.0045	0.0512 ± 0.0012
PEP40:5	C_45_H_80_O_7_NP	778.5750	(18:0p_22:5)				0.1258 ± 0.0061	0.1077 ± 0.0018
Sum PEP							6.4627 ± 0.2098 ^b^	13.4478 ± 0.1300 ^a^
PE		[M+H]^+^						
PE32:1	C_37_H_72_O_8_NP	690.5074	(18:1_14:0)	(16:0_16:1)			0.0063 ± 0.0005	0.0070 ± 0.0010
PE33:2	C_38_H_72_O_8_NP	702.5074	(15:0_18:2)				0.0255 ± 0.0016	0.0679 ± 0.0018
PE33:1	C_38_H_74_O_8_NP	704.5230	(15:0_18:1)				0.0109 ± 0.0008	0.0211 ± 0.0003
PE34:3	C_39_H_72_O_8_NP	714.5074	(16:1_18:2)				0.1019 ± 0.0018	0.2325 ± 0.0022
PE34:2	C_39_H_74_O_8_NP	716.5230	(16:0_18:2)				0.4254 ± 0.0080	0.8300 ± 0.0010
PE34:1	C_39_H_76_O_8_NP	718.5387	(16:0_18:1)				0.1355 ± 0.0010	0.1107 ± 0.0038
PE35:3	C_40_H_74_O_8_NP	728.5230	(17:1_18:2)				0.0236 ± 0.0008	0.0576 ± 0.0013
PE35:2	C_40_H_76_O_8_NP	730.5387	(17:0_18:2)				0.0773 ± 0.0018	0.1439 ± 0.0048
PE35:1	C_40_H_78_O_8_NP	732.5543	(17:0_18:1)				0.0359 ± 0.0014	0.0509 ± 0.0017
PE36:6	C_41_H_70_O_8_NP	736.4917	(18:3_18:3)				0.0075 ± 0.0025	0.0105 ± 0.0027
PE36:5	C_41_H_72_O_8_NP	738.5074	(18:3_18:2)				0.1666 ± 0.0068	0.3224 ± 0.0077
PE36:4	C_41_H_74_O_8_NP	740.5230	(18:4_18:0)				0.3182 ± 0.0054	0.5095 ± 0.0097
PE36:3	C_41_H_76_O_8_NP	742.5387	(18:1_18:2)				0.7075 ± 0.0043	1.0654 ± 0.0147
PE36:2	C_41_H_78_O_8_NP	744.5543	(18:0_18:2)				1.3071 ± 0.0211	2.1185 ± 0.0245
PE36:1	C_41_H_80_O_8_NP	746.5700	(17:1_19:0)	(18:0_18:1)			0.2672 ± 0.0048	0.3017 ± 0.0027
PE37:4	C_42_H_76_O_8_NP	754.5387	(19:1_18:3)				0.0209 ± 0.0006	0.0423 ± 0.0044
PE37:2	C_42_H_80_O_8_NP	758.5700	(19:0_18:2)				0.0139 ± 0.0030	0.0286 ± 0.0004
PE37:1	C_42_H_82_O_8_NP	760.5856	(19:0_18:1)				0.0062 ± 0.0005	0.0116 ± 0.0001
PE37:0	C_42_H_84_O_8_NP	762.6013	(16:0_21:0)				0.0036 ± 0.0006	0.006 ± 0.0006
PE38:7	C_43_H_72_O_8_NP	762.5074	(18:2_20:5	(18:3_20:4)			0.0211 ± 0.0013	0.0292 ± 0.0010
PE38:6	C_43_H_74_O_8_NP	764.5230	(16:1_22:5)	(16:0_22:6)	(18:1_20:5)		0.1360 ± 0.0105	0.1182 ± 0.0058
PE38:5	C_43_H_76_O_8_NP	766.5387	(16:0_22:5)	(18:2_20:3)			0.4967 ± 0.0168	0.6771 ± 0.0062
PE38:4	C_43_H_78_O_8_NP	768.5543	(18:1_20:3)				0.4559 ± 0.0167	0.6417 ± 0.0035
PE38:3	C_43_H_80_O_8_NP	770.5700	(20:0_18:3)				0.0672 ± 0.0031	0.1725 ± 0.0047
PE38:2	C_43_H_82_O_8_NP	772.5856	(20:0_18:2)				0.0739 ± 0.0032	0.1086 ± 0.0067
PE38:1	C_43_H_84_O_8_NP	774.6013	(17:1_21:0)	(20:0_18:1)			0.1706 ± 0.0058	0.1514 ± 0.0025
PE39:6	C_44_H_76_O_8_NP	778.5387	(18:3_21:3)	(16:1_23:5)			0.0225 ± 0.0027	0.0181 ± 0.0023
PE39:5	C_44_H_78_O_8_NP	780.5543	(18:3_21:2)				0.0152 ± 0.0012	0.0267 ± 0.0004
PE39:4	C_44_H_80_O_8_NP	782.5700	(19:0_20:4)	(16:0_23:4)	(18:3_21:1)		0.0029 ± 0.0010	0.0091 ± 0.0004
PE39:3	C_44_H_82_O_8_NP	784.5856	(18:3_21:0)	(18:2_21:1)			0.0006 ± 0.0001	0.0039 ± 0.0007
PE40:6	C_45_H_78_O_8_NP	792.5543	(18:1_22:5)				0.0733 ± 0.0026	0.0430 ± 0.0032
PE40:5	C_45_H_80_O_8_NP	794.5700	(18:0_22:5)				0.1507 ± 0.0029	0.1851 ± 0.0028
PE40:2	C_45_H_86_O_8_NP	800.6169	(18:2_22:0)				0.0033 ± 0.0004	0.0076 ± 0.0006
PE40:1	C_45_H_88_O_8_NP	802.6326	(19:1_21:0)				0.0039 ± 0.0003	0.0121 ± 0.0009
Sum PE							5.3548 ± 0.1153 ^b^	8.1423 ± 0.0925 ^a^
LPE		[M+H]^+^						
LPE14:0	C_19_H_40_O_7_NP	426.2621	(14:0)				0.0012 ± 0.0001	0.0039 ± 0.0004
LPE15:0	C_20_H_42_O_7_NP	440.2777	(15:0)				0.0208 ± 0.0007	0.0425 ± 0.0006
LPE16:1	C_21_H_42_O_7_NP	452.2777	(16:1)				0.0150 ± 0.0004	0.0122 ± 0.0106
LPE16:0	C_21_H_44_O_7_NP	454.2934	(16:0)				0.0976 ± 0.0018	0.1205 ± 0.0024
LPE17:0	C_22_H_46_O_7_NP	468.3090	(17:0)				0.0150 ± 0.0010	0.0198 ± 0.0006
LPE18:3	C_23_H_42_O_7_NP	476.2777	(18:3)				0.1409 ± 0.0008	0.2367 ± 0.0017
LPE18:2	C_23_H_44_O_7_NP	478.2933	(18:2)				0.6413 ± 0.0020	0.9915 ± 0.0193
LPE18:1	C_23_H_46_O_7_NP	480.3090	(18:1)				0.3105 ± 0.0023	0.2955 ± 0.0075
LPE18:0	C_23_H_48_O_7_NP	482.3247	(18:0)				0.2268 ± 0.0019	0.2488 ± 0.0055
LPE20:5	C_25_H_42_O_7_NP	500.2777	(20:5)				0.0743 ± 0.0085	0.1353 ± 0.0079
Sum LPE							1.5436 ± 0.0101 ^b^	2.1067 ± 0.0548 ^a^
PCP		[M+H]^+^						
PCP30:0	C_38_H_76_O_7_NP	690.5438	(16:0p_14:0)				1.0459 ± 0.0208	4.6225 ± 0.1847
PCP32:3	C_40_H_74_O_7_NP	712.5281	(14:0p_18:3)				0.5097 ± 0.0369	2.3374 ± 0.0812
PCP32:2	C_40_H_76_O_7_NP	714.5438	(14:0p_18:2)				2.9845 ± 0.0529	13.5516 ± 0.5208
PCP32:1	C_40_H_78_O_7_NP	716.5594	(14:0p_18:1)	(16:1p_16:0)			3.1137 ± 0.0833	9.8405 ± 0.3293
PCP33:3	C_41_H_76_O_7_NP	726.5437	(12:0p_21:3)				1.4286 ± 0.0316	7.3460 ± 0.2868
PCP33:2	C_41_H_78_O_7_NP	728.5594	(12:0p_21:2)				7.6677 ± 0.0876	38.9074 ± 1.6968
PCP33:1	C_41_H_80_O_7_NP	730.5750	(20:0p_13:1)				4.7964 ± 0.0422	17.0722 ± 0.8424
PCP34:4	C_42_H_76_O_7_NP	738.5437	(16:1p_18:3)				0.8963 ± 0.0326	3.6547 ± 0.0847
PCP34:3	C_42_H_78_O_7_NP	740.5594	(16:1p_18:2)	(16:0p_18:3)			4.1764 ± 0.0343	18.8963 ± 0.7064
PCP34:2	C_42_H_80_O_7_NP	742.5750	(16:0p_18:2)	(16:1p_18:1)			7.7849 ± 0.1239	21.5978 ± 0.7924
PCP34:1	C_42_H_82_O_7_NP	744.5907	(16:0p_18:1)				10.0563 ± 0.0732	35.4665 ± 1.2953
PCP34:0	C_42_H_84_O_7_NP	746.6064	(16:0p_18:0)				3.0220 ± 0.0679	6.8326 ± 0.2801
PCP34:0e	C_42_H_86_O_7_NP	748.6220	(18:0e_16:0)				0.1131 ± 0.0159	0.4825 ± 0.0106
PCP35:5	C_43_H_76_O_7_NP	750.5437	(12:0p_23:5)				0.3946 ± 0.0089	1.6411 ± 0.0602
PCP35:2	C_43_H_82_O_7_NP	756.5907	(14:0p_21:2)	(18:2p_17:0)			1.8426 ± 0.0399	5.1415 ± 0.0592
PCP35:1	C_43_H_84_O_7_NP	758.6063	(14:0p_21:1)				0.9376 ± 0.0208	2.3790 ± 0.0709
PCP36:4	C_44_H_80_O_7_NP	766.5750	(16:0p_20:4)				3.0664 ± 0.0773	9.0941 ± 0.3265
PCP36:3	C_44_H_82_O_7_NP	768.5907	(16:0p_20:3)				3.4833 ± 0.0882	10.9291 ± 0.5581
PCP36:2	C_44_H_84_O_7_NP	770.6063	(18:0p_18:2)	(18:2p_18:0)			3.4091 ± 0.0091	7.9785 ± 0.2539
PCP36:1	C_44_H_86_O_7_NP	772.6220	(18:0p_18:1)				2.6497 ± 0.0574	5.5222 ± 0.1870
PCP36:1e	C_44_H_88_O_7_NP	774.6376	(18:0e_18:1)				0.3786 ± 0.0191	0.8402 ± 0.0555
PCP37:5	C_45_H_80_O_7_NP	778.5751	(17:0p_20:5)				1.9257 ± 0.0132	4.8124 ± 0.2339
PCP38:5	C_46_H_82_O_7_NP	792.5907	(18:1p_20:4)	(16:1p_22:4)	(16:0p_22:5)		2.6230 ± 0.0785	5.0838 ± 0.2547
PCP38:4	C_46_H_84_O_7_NP	794.6064	(18:1p_20:3)				1.5538 ± 0.0339	3.3758 ± 0.1299
Sum PCP							69.8598 ± 0.6163 ^b^	237.4056 ± 8.7765 ^a^
PC		[M+H]^+^						
PC28:1	C_36_H_70_O_8_NP	676.4917	(14:0_14:1)	(16:0_12:1)	(10:0_18:1)		0.0015 ± 0.0002	0.0047 ± 0.0004
PC28:0	C_36_H_72_O_8_NP	678.5083	(16:0_12:0)				0.0071 ± 0.0006	0.0442 ± 0.0022
PC29:0	C_37_H_74_O_8_NP	692.5239	(14:0_15:0)				0.0129 ± 0.0008	0.1301 ± 0.0025
PC30:2	C_38_H_72_O_8_NP	702.5074	(16:0_14:2)	(12:0_18:2)			0.0040 ± 0.0010	0.0162 ± 0.0024
PC30:1	C_38_H_74_O_8_NP	704.5239	(16:0_14:1)				0.0451 ± 0.0010	0.1204 ± 0.0150
PC30:0	C_38_H_76_O_8_NP	706.5396	(16:0_14:0)				0.2832 ± 0.0049	1.3272 ± 0.0415
PC31:1	C_39_H_76_O_8_NP	718.5396	(17:1_14:0)	(15:0_16:1)			0.1616 ± 0.0037	0.6787 ± 0.0081
PC31:0	C_39_H_78_O_8_NP	720.5552	(16:0_15:0)	(14:0_17:0)			0.7876 ± 0.0206	4.3283 ± 0.0743
PC32:3	C_40_H_74_O_8_NP	728.5230	(14:1_18:2)				0.0568 ± 0.0138	0.2641 ± 0.0205
PC32:2	C_40_H_76_O_8_NP	730.5396	(14:0_18:2)	(16:1_16:1)			0.4550 ± 0.0024	1.4316 ± 0.0047
PC32:1	C_40_H_78_O_8_NP	732.5552	(16:0_16:1)				3.5580 ± 0.0531	6.7597 ± 0.0945
PC32:0	C_40_H_80_O_8_NP	734.5709	(16:0_16:0)				2.8296 ± 0.0759	7.0457 ± 0.1375
PC33:3	C_41_H_76_O_8_NP	742.5396	(15:0_18:3)				0.3355 ± 0.0151	1.9368 ± 0.0121
PC33:2	C_41_H_78_O_8_NP	744.5552	(15:0_18:2)				2.3396 ± 0.0261	9.8380 ± 0.1481
PC33:1	C_41_H_80_O_8_NP	746.5709	(17:1_16:0)	(15:0_18:1)			4.6588 ± 0.067	11.5965 ± 0.1967
PC33:0	C_41_H_82_O_8_NP	748.5865	(16:0_17:0)				0.8003 ± 0.0241	3.8705 ± 0.0143
PC34:6	C_42_H_72_O_8_NP	750.5074	(18:2_20:4)				0.0020 ± 0.0003	0.0096 ± 0.0079
PC34:5	C_42_H_74_O_8_NP	752.5230	(14:0_20:5)				0.0392 ± 0.0037	0.1057 ± 0.0016
PC34:4	C_42_H_76_O_8_NP	754.5387	(14:0_20:4)				0.5894 ± 0.0344	1.3508 ± 0.0353
PC34:3	C_42_H_78_O_8_NP	756.5552	(16:0_18:3)	(16:1_18:2)			12.9280 ± 0.2866	35.8247 ± 0.6425
PC34:2	C_42_H_80_O_8_NP	758.5709	(16:0_18:2)				72.3397 ± 1.4917	164.5289 ± 2.0029
PC34:1	C_42_H_82_O_8_NP	760.5865	(16:0_18:1)				72.7828 ± 1.4208	95.9041 ± 1.5406
PC34:0	C_42_H_84_O_8_NP	762.6022	(18:0_16:0)				0.0288 ± 0.0031	0.3867 ± 0.0560
PC35:3	C_43_H_80_O_8_NP	770.5709	(17:0_18:3)				2.1995 ± 0.0245	9.2430 ± 0.1707
PC35:2	C_43_H_82_O_8_NP	772.5865	(17:0_18:2)	(17:1_18:1)			8.5158 ± 0.1864	30.6083 ± 0.4743
PC35:1	C_43_H_84_O_8_NP	774.6022	(17:0_18:1)	(17:1_18:0)			8.0647 ± 0.1882	18.7384 ± 0.1406
PC35:0	C_43_H_86_O_8_NP	776.6178	(18:0_17:0)				0.0795 ± 0.0052	0.3570 ± 0.0588
PC36:6	C_44_H_76_O_8_NP	778.5387	(18:3_18:3)				0.2713 ± 0.0208	0.5305 ± 0.0449
PC36:5	C_44_H_78_O_8_NP	780.5552	(18:3_18:2)	(22:4_14:1)	(16:0_20:5)		4.0667 ± 0.1229	8.3473 ± 0.1490
PC36:4	C_44_H_80_O_8_NP	782.5709	(18:2_18:2)	(18:1_18:3)	(16:0_20:4)		16.6778 ± 0.3539	34.0483 ± 0.4082
PC36:3	C_44_H_82_O_8_NP	784.5865	(18:1_18:2)	(18:0_18:3)			49.9707 ± 1.3100	109.1497 ± 0.9252
PC36:2	C_44_H_84_O_8_NP	786.6022	(18:0_18:2)	(18:1_18:1)			110.6408 ± 2.4982	252.3240 ± 2.9393
PC36:1	C_44_H_86_O_8_NP	788.6178	(18:0_18:1)				80.7553 ± 1.6176	129.9403 ± 0.7620
PC36:0	C_44_H_88_O_8_NP	790.6335	(20:0_16:0)				1.4918 ± 0.1599	3.1499 ± 0.4775
PC37:4	C_45_H_82_O_8_NP	796.5856	(17:0_20:4)				1.1948 ± 0.0139	3.7518 ± 0.0208
PC37:3	C_45_H_84_O_8_NP	798.6022	(19:1_18:2)	(19:2_18:1)			1.1953 ± 0.0445	4.6386 ± 0.0911
PC37:2	C_45_H_86_O_8_NP	800.6178	(19:0_18:2)	(19:1_18:1)			1.0680 ± 0.0268	3.2745 ± 0.1055
PC37:1	C_45_H_88_O_8_NP	802.6335	(19:0_18:1)	(19:1_18:0)			0.9939 ± 0.0282	3.2822 ± 0.0265
PC37:0	C_45_H_90_O_8_NP	804.6491	(20:0_17:0)				0.4192 ± 0.0245	1.2088 ± 0.0640
PC38:7	C_46_H_78_O_8_NP	804.5543	(18:2_20:5)				0.3546 ± 0.0047	0.6432 ± 0.0141
PC38:6	C_46_H_80_O_8_NP	806.5709	(18:1_20:5)	(18:2_20:4)			4.0472 ± 0.1119	4.2582 ± 0.0541
PC38:5	C_46_H_82_O_8_NP	808.5865	(18:0_20:5)	(18:1_20:4)	(16:0_22:5)		18.5891 ± 0.5608	28.4122 ± 0.3486
PC38:4	C_46_H_84_O_8_NP	810.6022	(18:1_20:3)	(18:0_20:4)	(20:2_18:2)		19.9458 ± 0.5601	40.5747 ± 0.4657
PC38:3	C_46_H_86_O_8_NP	812.6178	(20:0_18:3)	(18:0_20:3)			18.6136 ± 0.5430	57.4257 ± 0.5301
PC38:2	C_46_H_88_O_8_NP	814.6335	(18:0_20:2)	(20:0_18:2)			24.67786 ± 0.6280	94.1431 ± 1.1454
PC38:1	C_46_H_90_O_8_NP	816.6491	(18:0_20:1)				3.9525 ± 0.1459	5.103 ± 0.1656
PC38:0	C_46_H_92_O_8_NP	818.6648	(18:0_20:0)				2.1885 ± 0.1293	4.5270 ± 0.1179
PC40:7	C_48_H_82_O_8_NP	832.5865	(22:5_18:2)	(18:1_22:6)	(20:3_20:4)		1.1093 ± 0.0509	0.9485 ± 0.0403
PC40:6	C_48_H_84_O_8_NP	834.6022	(18:0_22:6)	(18:1_22:5)	(22:4_18:2)		6.8078 ± 0.2098	4.4424 ± 0.0460
PC40:5	C_48_H_86_O_8_NP	836.6178	(18:0_22:5)	(20:0_20:5)	(20:2_20:3)	(18:1_22:4)	19.377 ± 0.5128	33.4054 ± 0.3633
PC40:4	C_48_H_88_O_8_NP	838.6335	(20:0_20:4)	(18:0_22:4)			3.2918 ± 0.1870	25.7167 ± 0.3363
PC40:3	C_48_H_90_O_8_NP	840.6491	(20:0_20:3)				1.3870 ± 0.0605	8.4191 ± 2.0322
PC40:1	C_48_H_94_O_8_NP	844.6795	(20:0_20:1)				0.0176 ± 0.0018	0.0856 ± 0.0198
PC42:5	C_50_H_90_O_8_NP	864.6491	(20:0_22:5)				0.9225 ± 0.0246	4.2514 ± 0.1122
Sum PC							588.5461 ± 12.9369 ^b^	1272.464 ± 13.0259 ^a^
SM		[M+H]^+^						
SM30:1	C_35_H_71_O_6_N_2_P	647.5128	(d18:1_12:0)	(d16:1_14:0)			0.1283 ± 0.0018	0.3177 ± 0.0049
SM31:1	C_36_H_73_O_6_N_2_P	661.5284	(d17:1_14:0)				0.1335 ± 0.0024	0.3959 ± 0.0100
SM32:2	C_37_H_73_O_6_N_2_P	673.5284	(d16:1_16:1)	(d18:2_14:0)			0.1368 ± 0.0028	0.3696 ± 0.0064
SM32:1	C_37_H_75_O_6_N_2_P	675.5441	(d16:1_16:0)	(d18:1_14:0)	(d17:1_15:0)		3.3529 ± 0.0679	7.8838 ± 0.0363
SM32:0	C_37_H_77_O_6_N_2_P	677.5597	(d16:0_16:0)	(d18:0_14:0)			0.2728 ± 0.0056	0.5612 ± 0.0071
SM33:2	C_38_H_75_O_6_N_2_P	687.5441	(d18:2_15:0)	(d17:1_16:1)			0.3111 ± 0.0035	1.0026 ± 0.0049
SM33:1	C_38_H_77_O_6_N_2_P	689.5597	(d15:0_18:1)	(d17:1_16:0)	(d18:1_15:0)		7.1329 ± 0.1758	13.6523 ± 0.0685
SM34:4	C_39_H_73_O_6_N_2_P	697.5284	(d16:1_18:3)	(d16:0_18:4)			0.2832 ± 0.0268	0.9461 ± 0.0227
SM34:2	C_39_H_77_O_6_N_2_P	701.5597	(d16:1_18:1)	(d18:2_16:0)	(d18:1_16:1)		5.7788 ± 0.0882	11.7693 ± 0.1712
SM34:1	C_39_H_79_O_6_N_2_P	703.5754	(d17:1_17:0)	(d16:0_18:1)	(d18:1_16:0)		59.6131 ± 1.1267	109.9824 ± 0.5397
SM34:0	C_39_H_81_O_6_N_2_P	705.5910	(d16:0_18:0)				2.4911 ± 0.1915	3.6841 ± 0.3210
SM35:2	C_40_H_79_O_6_N_2_P	715.5754	(d18:2_17:0)	(d18:1_17:1)			0.5853 ± 0.0197	1.9068 ± 0.0371
SM35:1	C_40_H_81_O_6_N_2_P	717.5910	(d17:0_18:1)	(d16:0_19:1)	(d18:1_17:0)		5.2828 ± 0.1255	13.5006 ± 0.0116
SM36:2	C_41_H_81_O_6_N_2_P	729.5910	(d18:0_18:2)				2.6242 ± 0.0514	4.8988 ± 0.0770
SM36:1	C_41_H_83_O_6_N_2_P	731.6067	(d18:0_18:1)	(d18:1_18:0)			6.4462 ± 0.1605	13.5778 ± 0.0486
SM37:1	C_42_H_85_O_6_N_2_P	745.6223	(d19:1_18:0)				0.6886 ± 0.0294	1.4944 ± 0.0035
SM38:2	C_43_H_85_O_6_N_2_P	757.6223	(d20:0_18:2)				0.2087 ± 0.0084	0.3746 ± 0.0169
SM38:1	C_43_H_87_O_6_N_2_P	759.6380	(d20:0_18:1)	(d18:1_20:0)			1.1067 ± 0.0317	1.7807 ± 0.0088
SM39:2	C_44_H_87_O_6_N_2_P	771.6380	(d21:0_18:2)				0.3146 ± 0.0070	0.5677 ± 0.0099
SM39:1	C_44_H_89_O_6_N_2_P	773.6536	(d16:0_23:1)	(d16:1_23:0)	(d17:1_22:0)		1.9471 ± 0.0576	3.2459 ± 0.0204
SM40:3	C_45_H_87_O_6_N_2_P	783.6380	(d22:0_18:3)				0.2064 ± 0.0082	0.3284 ± 0.0166
SM40:2	C_45_H_89_O_6_N_2_P	785.6536	(d16:0_24:2)	(d22:0_18:2)			2.1756 ± 0.0797	3.5461 ± 0.0217
SM40:1	C_45_H_91_O_6_N_2_P	787.6693	(d20:1_20:0)	(d16:0_24:1)	(d18:1_22:0)	(d16:1_24:0)	5.8898 ± 0.1823	8.8262 ± 0.1028
SM40:0	C_45_H_93_O_6_N_2_P	789.6849	(d16:0_24:0)	(d20:0_20:0)			0.0222 ± 0.0008	0.0374 ± 0.0116
SM41:3	C_46_H_89_O_6_N_2_P	797.6536	(d18:2_23:1)	(d18:1_23:2)			0.4247 ± 0.0149	0.7296 ± 0.0110
SM41:2	C_46_H_91_O_6_N_2_P	799.6693	(d17:0_24:2)	(d16:0_25:2)	(d18:2_23:0)	(d17:1_24:1)	3.4975 ± 0.1228	5.8030 ± 0.0584
SM41:1	C_46_H_93_O_6_N_2_P	801.6849	(d16:0_25:1)	(d18:1_23:0)	(d17:1_24:0)		7.4893 ± 0.2721	11.9295 ± 0.0572
SM42:3	C_47_H_91_O_6_N_2_P	811.6693	(d16:0_26:3)	(d18:2_24:1)	(d18:1_24:2)		1.4378 ± 0.0499	2.4176 ± 0.0072
SM42:2	C_47_H_93_O_6_N_2_P	813.6849	(d16:0_26:2)	(d18:1_24:1)	(d18:2_24:0)		5.3595 ± 0.1935	8.3947 ± 0.0560
SM42:1	C_47_H_95_O_6_N_2_P	815.7006	(d16:0_26:1)	(d18:1_24:0)			4.9400 ± 0.1953	8.1677 ± 0.1511
SM43:4	C_48_H_91_O_6_N_2_P	823.6693	(d20:1_23:3)				3.9681 ± 0.0922	5.5671 ± 0.0337
SM43:2	C_48_H_95_O_6_N_2_P	827.7006	(d16:0_27:2)	(d18:1_25:1)	(d18:2_25:0)		0.8206 ± 0.0408	1.6176 ± 0.0170
SM43:1	C_48_H_97_O_6_N_2_P	829.7162	(d16:0_27:1)	(d18:1_25:0)			0.7480 ± 0.0257	1.4972 ± 0.0078
SM44:5	C_49_H_91_O_6_N_2_P	835.6693	(d18:1_26:4)				4.1182 ± 0.1350	5.4686 ± 0.0546
SM44:4	C_49_H_93_O_6_N_2_P	837.6849	(d18:1_26:1)				2.9638 ± 0.1961	4.4989 ± 0.0238
SM44:2	C_49_H_97_O_6_N_2_P	841.7162	(d16:0_28:2)	(d18:2_26:0)			0.2070 ± 0.0106	0.3471 ± 0.0002
SM44:1	C_49_H_99_O_6_N_2_P	843.7319	(d16:0_28:1)	(d18:1_26:0)			0.1117 ± 0.0035	0.1947 ± 0.0033
Sum SM							143.2188 ± 3.6077 ^b^	261.2838 ± 0.8030 ^a^
LPC		[M+H]^+^						
LPC14:0	C_22_H_46_O_7_NP	468.309	(14:0)				0.2287 ± 0.0033	0.6070 ± 0.0073
LPC15:0	C_23_H_48_O_7_NP	482.3246	(15:0)				1.0510 ± 0.0154	1.9795 ± 0.0236
LPC16:2	C_24_H_46_O_7_NP	492.3090	(16:2)				0.0463 ± 0.0004	0.0539 ± 0.0020
LPC16:1	C_24_H_48_O_7_NP	494.3246	(16:1)				1.3762 ± 0.0261	1.3727 ± 0.0167
LPC16:0	C_24_H_50_O_7_NP	496.3403	(16:0)				27.1484 ± 0.6959	26.7757 ± 0.2697
LPC17:1	C_25_H_50_O_7_NP	508.3403	(17:1)				0.5561 ± 0.0193	1.1146 ± 0.0100
LPC17:0	C_25_H_52_O_7_NP	510.3559	(17:0)				2.4971 ± 0.0521	4.3784 ± 0.0398
LPC18:3	C_26_H_48_O_7_NP	518.3246	(18:3)				1.6941 ± 0.2449	4.2941 ± 0.1436
LPC18:2	C_26_H_50_O_7_NP	520.3403	(18:2)				9.7862 ± 0.3058	19.0173 ± 0.5417
LPC18:1	C_26_H_52_O_7_NP	522.3559	(18:1)				16.2924 ± 0.4021	17.0299 ± 0.3764
LPC18:0	C_26_H_54_O_7_NP	524.3716	(18:0)				28.5778 ± 0.5239	33.5810 ± 0.4548
LPC19:1	C_27_H_54_O_7_NP	536.3716	(19:1)				0.1213 ± 0.0043	0.2568 ± 0.0093
LPC20:5	C_28_H_48_O_7_NP	542.3246	(20:5)				0.0152 ± 0.0049	0.0210 ± 0.0033
LPC20:4	C_28_H_50_O_7_NP	544.3403	(20:4)				0.0595 ± 0.0188	0.1922 ± 0.0260
LPC20:3	C_28_H_52_O_7_NP	546.3559	(20:3)				0.0092 ± 0.0053	0.1485 ± 0.0353
LPC20:1	C_28_H_56_O_7_NP	550.3873	(20:1)				0.0866 ± 0.0021	0.1084 ± 0.0030
LPC20:0	C_28_H_58_O_7_NP	552.4029	(20:0)				0.8115 ± 0.0376	1.8312 ± 0.0681
LPC22:6	C_30_H_50_O_7_NP	568.3403	(22:6)				0.2215 ± 0.0088	0.1701 ± 0.0069
LPC22:5	C_30_H_52_O_7_NP	570.3559	(22:5)				0.7470 ± 0.0214	0.8575 ± 0.0397
Sum LPC							91.3260 ± 2.2802 ^b^	113.7898 ± 1.8506 ^a^
PS		[M+H]^+^						
PS 36:3	C_42_H_76_O_10_NP	786.5291	(18:1_18:2)				0.0223 ± 0.0010	0.0203 ± 0.0011
PS 36:2	C_42_H_78_O_10_NP	788.5447	(18:0_18:2)				0.0986 ± 0.0037	0.077 ± 0.0017
PS 36:1	C_42_H_80_O_10_NP	790.5604	(18:0_18:1)				0.0798 ± 0.0021	0.0607 ± 0.0014
PS 38:4	C_44_H_78_O_10_NP	812.5447	(18:1_20:3)				0.1214 ± 0.0030	0.0971 ± 0.0035
PS 38:3	C_44_H_80_O_10_NP	814.5604	(18:0_20:3)				0.0198 ± 0.0005	0.0491 ± 0.0032
PS 40:5	C_46_H_80_O_10_NP	838.5604	(18:0_22:5)				0.0814 ± 0.0030	0.0904 ± 0.0011
Sum PS							0.4234 ± 0.0037 ^a^	0.3947 ± 0.0094 ^a^
PA		[M-H]^−^						
PA 34:2	C_37_H_69_O_8_P	671.4657	(16:0_18:2)				0.0514 ± 0.0013	0.0646 ± 0.0015
PA 34:1	C_37_H_71_O_8_P	673.4814	(16:0_18:1)				0.052 ± 0.0030	0.035 ± 0.0026
PA 36:3	C_39_H_71_O_8_P	697.4814	(18:2_18:1)	(18:0_18:3)			0.0334 ± 0.0033	0.0563 ± 0.0068
PA 36:2	C_39_H_73_O_8_P	699.4970	(18:2_18:0)				0.0895 ± 0.0031	0.124 ± 0.0061
PA 36:1	C_39_H_75_O_8_P	701.5127	(18:0_18:1)				0.0474 ± 0.0062	0.052 ± 0.0035
Sum PA							0.2736 ± 0.0109 ^b^	0.3319 ± 0.0136 ^a^
PG		[M-H]^−^						
PG 36:4	C_42_H_75_O_10_P	769.5026	(18:2_18:2)				0.0002 ± 0.0002	0.002 ± 0.0008
PG 36:3	C_42_H_77_O_10_P	771.5182	(18:2_18:1)				0.0006 ± 0.0005	0.002 ± 0.0004
PG 36:2	C_42_H_79_O_10_P	773.5338	(18:2_18:0)	(18:1_18:1)			0.0037 ± 0.0006	0.0052 ± 0.0012
PG 36:1	C_42_H_81_O_10_P	775.5495	(18:1_18:0)				0.0049 ± 0.0007	0.0052 ± 0.0010
Sum PG							0.0095 ± 0.0013 ^b^	0.0144 ± 0.0008 ^a^
AcylCar		[M+H]^+^						
CAR 2:0	C_9_H_17_NO_4_	204.1237	(2:0)				0.3670 ± 0.0107	0.1514 ± 0.0015
CAR 3:0	C_10_H_19_NO_4_	218.1393	(3:0)				0.0422 ± 0.0007	0.0406 ± 0.0009
CAR 4:0	C_11_H_21_NO_4_	232.1549	(4:0)				0.0213 ± 0.0005	0.0153 ± 0.0004
CAR 5:0	C_12_H_23_NO_4_	246.1705	(5:0)				0.0142 ± 0.0001	0.0145 ± 0.0001
CAR 6:0	C_13_H_25_NO_4_	260.1861	(6:0)				0.0028 ± 0.0002	0.0016 ± 0.0002
CAR 8:0	C_15_H_29_NO_4_	288.2175	(8:0)				0.0005 ± 0.0001	0.0002 ± 0.0000
CAR 10:0	C_17_H_33_NO_4_	316.2488	(10:0)				0.0010 ± 0.0001	0.0003 ± 0.0000
CAR 14:0	C_21_H_41_NO_4_	372.3114	(14:0)				0.0079 ± 0.0014	0.0044 ± 0.0002
CAR 16:0	C_23_H_45_NO_4_	400.3427	(16:0)				0.0093 ± 0.0006	0.0010 ± 0.0001
CAR 18:0	C_25_H_49_NO_4_	428.3740	(18:0)				0.0224 ± 0.0006	0.0065 ± 0.0003
CAR 18:1	C_25_H_47_NO_4_	426.3583	(18:1)				0.0177 ± 0.0004	0.0028 ± 0.0001
CAR 20:0	C_27_H_53_NO_4_	456.4047	(20:0)				0.0013 ± 0.0001	0.0010 ± 0.0002
Sum AcylCar							0.5076 ± 0.0109 ^a^	0.2397 ± 0.0010 ^b^
CE		[M+NH_4_]^+^						
CE 16:0	C_43_H_76_O_2_	642.6189	16:0				9.2963 ± 0.2254	18.8677 ± 0.7220
CE 18:4	C_45_H_72_O_2_	662.5876	18:4				25.2081 ± 0.1739	112.3138 ± 2.5923
CE 18:3	C_45_H_74_O_2_	664.6033	18:3				1111.8632 ± 32.6934	2138.4252 ± 15.2436
CE 18:2	C_45_H_76_O_2_	666.6189	18:2				1382.3895 ± 0.6956	2388.5635 ± 26.2006
CE 18:1	C_45_H_78_O_2_	668.6346	18:1				86.3225 ± 3.4780	137.1509 ± 2.3149
CE 18:0	C_45_H_80_O_2_	670.6502	18:0				0.3935 ± 0.0174	2.0939 ± 0.0126
CE 20:5	C_47_H_74_O_2_	688.6033	20:5				222.4054 ± 4.3741	372.8921 ± 2.4270
CE 20:4	C_47_H_76_O_2_	690.6189	20:4				200.8453 ± 2.2851	366.8301 ± 2.3469
CE 20:3	C_47_H_78_O_2_	692.6346	20:3				44.5959 ± 0.1420	114.5029 ± 4.8012
CE 20:2	C_47_H_80_O_2_	694.6502	20:2				3.6234 ± 0.0376	9.4212 ± 0.3536
CE 22:6	C_49_H_76_O_3_	714.6183	22:6				27.2166 ± 0.7906	11.0798 ± 0.1913
Sum CE							3114.1596 ± 32.2322 ^b^	5672.1411 ± 36.3072 ^a^
Cer		[M+H]^+^						
Cer 34:1	C_34_H_67_O_3_N	538.5184	(d18:1_16:0)				0.0753 ± 0.0003	0.1140 ± 0.0053
Cer 36:1	C_36_H_71_O_3_N	566.5512	(d18:1_18:0)				0.0062 ± 0.0001	0.0098 ± 0.0010
Cer 38:1	C_38_H_75_O_3_N	594.5825	(d18:1_20:0)				0.0020 ± 0.0001	0.0030 ± 0.0011
Cer 39:1	C_39_H_77_O_3_N	608.5981	(d16:1_23:0)	(18:1_21:0)	(17:1_22:0)		0.0056 ± 0.0000	0.0086 ± 0.0008
Cer 39:0	C_39_H_79_O_3_N	610.6138	(d16:0_23:0)				0.0044 ± 0.0000	0.0058 ± 0.0004
Cer 40:1	C_40_H_79_O_3_N	622.6138	(d18:1_22:0)				0.0423 ± 0.0009	0.0476 ± 0.0025
Cer 40:0	C_40_H_81_O_3_N	624.6294	(d18:0_22:0)				0.0300 ± 0.0002	0.0399 ± 0.0088
Cer 41:1	C_41_H_81_O_3_N	636.6294	(d18:1_23:0)				0.1037 ± 0.0000	0.1189 ± 0.0028
Cer 41:0	C_41_H_83_O_3_N	638.6445	(d18:0_23:0)				0.0565 ± 0.0011	0.0835 ± 0.0073
Cer 42:2	C_42_H_81_O_3_N	648.6288	(d18:2_24:0)				0.0342 ± 0.0008	0.0382 ± 0.0052
Cer 42:1	C_42_H_83_O_3_N	650.6451	(d18:1_24:0)				0.1235 ± 0.0012	0.1291 ± 0.0057
Cer 42:0	C_42_H_85_O_3_N	652.6601	(d18:0_24:0)				0.0490 ± 0.0002	0.0732 ± 0.0074
Cer 43:1	C_43_H_85_O_3_N	664.6598	(d18:1_25:0)				0.0435 ± 0.0000	0.0520 ± 0.0029
Sum Cer							0.6144 ± 0.0096 ^b^	0.7867 ± 0.0479 ^a^
LacCer		[M+H]^+^						
LacCer 32:1	C_44_H_83_O_13_N	834.5937	(d16:1_16:0)	(d18:1_14:0)			0.0244 ± 0.0009	0.0647 ± 0.0027
LacCer 33:1	C_45_H_85_O_13_N	848.6094	(d18:1_15:0)				0.0282 ± 0.0006	0.0544 ± 0.0007
LacCer 34:2	C_46_H_85_O_13_N	860.6094	(d18:2_16:0)	(d18:1_16:1)	(d16:1_18:1)		0.0478 ± 0.0018	0.0571 ± 0.0030
LacCer 34:1	C_46_H_87_O_13_N	862.625	(d18:1_16:0)				0.7095 ± 0.0083	0.8106 ± 0.0158
LacCer 36:2	C_48_H_89_O_13_N	888.6407	(d18:1_18:1)				0.0076 ± 0.0004	0.0103 ± 0.0006
LacCer 36:1	C_48_H_91_O_13_N	890.6563	(d18:1_18:0)	(d16:1_20:0)			0.0193 ± 0.0013	0.0362 ± 0.0003
LacCer 37:1	C_49_H_93_O_13_N	904.6720	(d16:1_21:0)				0.0013 ± 0.0002	0.0033 ± 0.0003
LacCer 38:1	C_50_H_95_O_13_N	918.6876	(d16:1_22:0)				0.0039 ± 0.0003	0.0120 ± 0.0007
LacCer 39:1	C_51_H_97_O_13_N	932.7033	(d17:1_22:0)	(d18:1_21:0)	(d16:1_23:0)		0.0022 ± 0.0001	0.0089 ± 0.0002
LacCer 40:2	C_52_H_97_O_13_N	944.7033	(d18:1_22:1)				0.0051 ± 0.0004	0.0098 ± 0.0008
LacCer 40:1	C_52_H_99_O_13_N	946.7189	(d18:1_22:0)				0.0194 ± 0.0010	0.0410 ± 0.0020
LacCer 41:2	C_53_H_99_O_13_N	958.7189	(d18:2_23:0)	(d18:1_23:1)			0.0049 ± 0.0005	0.0117 ± 0.0004
LacCer 41:1	C_53_H_101_O_13_N	960.7351	(d18:1_23:0)				0.0115 ± 0.0000	0.0275 ± 0.0016
LacCer 42:2	C_54_H_101_O_13_N	972.7351	(d18:1_24:1)				0.0368 ± 0.0011	0.0480 ± 0.0013
LacCer 42:1	C_54_H_103_O_13_N	974.7508	(d18:1_24:0)				0.0184 ± 0.0002	0.0344 ± 0.0003
Sum LacCer							0.9402 ± 0.0107 ^b^	1.2299 ± 0.0231 ^a^
GluCer		[M+H]^+^						
GlcCer 34:1	C_40_H_77_O_8_N	700.5727	(d18:1_16:0)	(d16:0_18:1)			0.0499 ± 0.0012	0.0577 ± 0.0017
GlcCer 36:1	C_42_H_81_O_8_N	728.6035	(d18:1_18:0)	(d16:1_20:0)			0.0119 ± 0.0004	0.0159 ± 0.0004
GlcCer 38:1	C_44_H_85_O_8_N	756.6348	(d18:1_20:0)				0.0060 ± 0.0003	0.0093 ± 0.0006
GlcCer 39:1	C_45_H_87_O_8_N	770.6504	(d18:1_21:0)				0.0059 ± 0.0002	0.0110 ± 0.0006
GlcCer 40:1	C_46_H_89_O_8_N	784.6666	(d18:1_22:0)				0.0467 ± 0.0014	0.0740 ± 0.0021
GlcCer 41:1	C_47_H_91_O_8_N	798.6823	(d18:1_23:0)				0.0462 ± 0.0013	0.0904 ± 0.0023
GlcCer 42:1	C_48_H_93_O_8_N	812.6979	(d18:1_24:0)				0.0794 ± 0.0027	0.1309 ± 0.0048
Sum GluCer							0.2460 ± 0.0059 ^b^	0.3892 ± 0.0118 ^a^
FFA		[M-H]^−^						
C16:0	C_16_H_32_O_2_	255.2330	(16:0)				22.5066 ± 2.5411	5.8133 ± 0.7283
C18:0	C_18_H_36_O_2_	283.2643	(18:0)				29.2070 ± 3.7099	8.8109 ± 1.1622
C18:1	C_18_H_34_O_2_	281.2486	(18:1)				72.1494 ± 3.9841	21.1765 ± 0.7292
C18:2	C_18_H_32_O_2_	279.2330	(18:2)				14.0737 ± 0.5939	7.2478 ± 0.7854
C20:0	C_20_H_40_O_2_	311.2955	(20:0)				14.7507 ± 0.6851	8.4132 ± 0.4794
C20:1	C_20_H_38_O_2_	309.2799	(20:1)				0.5974 ± 0.0438	0.2718 ± 0.0852
Sum FFA							153.2849 ± 3.4000 ^a^	51.7336 ± 0.0853 ^b^
SCFA ^1^		[M-H]^−^	FA					
C2	C_2_H_4_O_2_	194.0566	(2:0)				50.6667 ± 0.1155	70.4000 ± 0.9165
C3	C_3_H_6_O_2_	208.0723	(3:0)				1.3037 ± 0.0296	2.2123 ± 0.0171
iso-C4	C_4_H_8_O_2_	222.0879	(4:0)				0.1462 ± 0.0014	0.2386 ± 0.0024
C4	C_4_H_8_O_2_	222.0879	(4:0)				0.8620 ± 0.0074	2.3270 ± 0.0147
Sum SCFA							52.9786 ± 0.1325 ^b^	75.1789 ± 0.9247 ^a^
BHBA	C_4_H_8_O_3_	238.0828	NA				81.1872 ± 1.9437 ^a^	76.3470 ± 0.5703 ^b^

Note: ^1^ For SCFA, *m*/*z* of 3-NPH-tagged molecules are given. All concentrations are means of 3 measurements ± SD of pooled samples from 15 cows for each stage. For each lipid class, the sum concentrations followed by different letters are significantly different between the two lactation stages (*p* < 0.05).

## Data Availability

Data available on request.

## Supplementary Materials

The following supporting information can be downloaded at: https://www.mdpi.com/article/10.3390/metabo15040274/s1, Figure S1: Comparison of the top 5 lipids between the two lactation stages. A: 2022 cohort (*n* = 143); B: 2023 cohort (*n* = 218). Error bars are standard deviation.
