# Supplementary material for: Comprehensive Characterization of Serum Lipids of Dairy Cows: Effects of Negative Energy Balance on Lipid Remodelling
Source: Metabolites. 2025 Apr 15;15(4):274. doi: 10.3390/metabo15040274 (PMC12029815; doi:10.3390/metabo15040274)
Supplement: Supplementary file 1 [file metabolites-15-00274-s001.zip › metabolites-3578820-supplementary.pptx]

## Slide 1
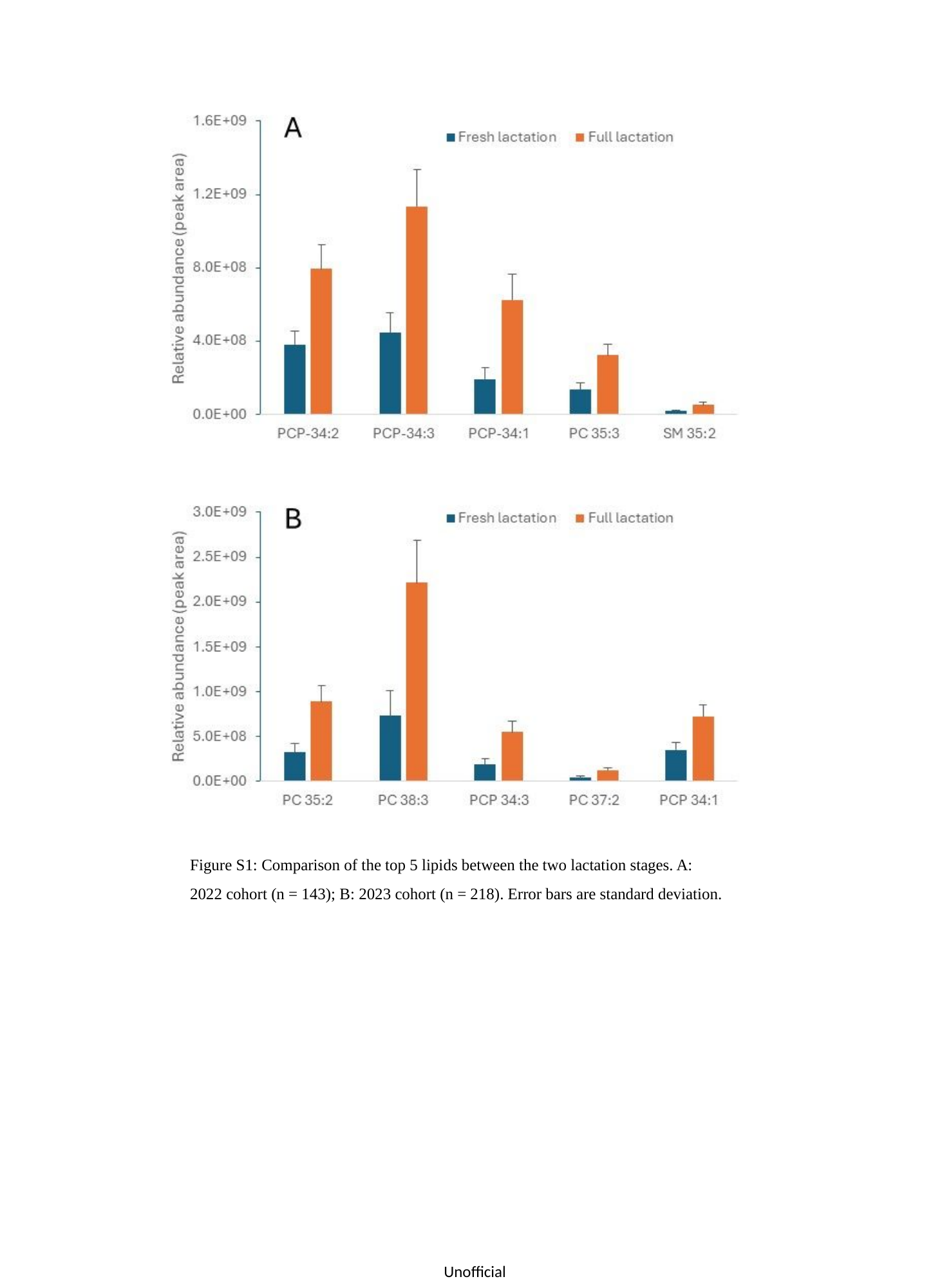

Figure S1: Comparison of the top 5 lipids between the two lactation stages. A: 2022 cohort (n = 143); B: 2023 cohort (n = 218). Error bars are standard deviation.
